# Equality of opportunity and mortality around the world: implications for global public health

**DOI:** 10.1080/16549716.2025.2540167

**Published:** 2025-08-20

**Authors:** Alexi Gugushvili, Elias Nosrati, Caspar Kaiser

**Affiliations:** a Department of Sociology and Human Geography, University of Oslo, Oslo, Norway; b Warwick Business School, The University of Warwick, Coventry, UK

**Keywords:** Equality of opportunity, educational mobility, all-cause mortality, intergenerational health disparities, global public health

## Abstract

**Background:**

Educational mobility is considered a key driver of population health. While prior studies suggest that intergenerational equality of opportunity may be linked to mortality, most evidence comes from high-income countries. Little is known about whether these associations apply globally.

**Objective:**

This study assesses the relationship between intergenerational educational mobility and all-cause mortality across a global sample of countries.

**Methods:**

We combine country-level data from the WHO Mortality Database and the World Bank’s Global Database on Intergenerational Mobility, covering five birth cohorts across 148 countries. Using multilevel random effects models, we estimate associations between four dimensions of educational mobility (upward, downward, stagnant, and correlation-based) and age- and sex-adjusted all-cause mortality, controlling for national indicators of education, income, inequality, health spending, unemployment, and political freedoms.

**Results:**

Higher upward educational mobility and lower stagnant mobility are significantly associated with reduced all-cause mortality. In fully adjusted models, a one standard deviation greater measure of upward mobility is associated with a reduction of 29.1 deaths per 100,000 population, while a one standard deviation lower stagnation measure is associated with a reduction of 27.3 deaths per 100,000 population. These patterns are consistent across high- and low-income countries.

**Conclusions:**

Our findings suggest that promoting educational equality of opportunity may reduce mortality and improve public health worldwide. Strengthening social mobility, especially in settings with persistent educational inequality, can be an effective policy lever for reducing health disparities and supporting healthier populations.

## Paper Context

**Main findings:** Higher upward educational mobility and lower stagnant mobility are significantly associated with reduced all-cause mortality across 148 countries and five cohorts;

**Added knowledge:** This study provides the first global evidence linking intergenerational educational mobility to mortality, expanding research beyond high-income countries;

**Global health impact for policy and action:** Promoting educational equality of opportunity can serve as an effective public health strategy to reduce mortality and improve population health worldwide.

## Background

Equality of opportunity refers to the principle that all individuals should have the same chances to succeed in life, regardless of their social background or other ascribed characteristics, such as gender, race, or ethnicity [1]. From an intergenerational perspective, the concept of equality of opportunity emphasizes the idea that all children should have the same life opportunities, regardless of their parents’ occupation, education, income, wealth, or social connections [2,3]. While there is an extensive literature on the consequences of intergenerational mobility on health and well-being outcomes at an individual level [4,5], only a handful of studies have investigated the links between equality of opportunity and health at a population level [6,7]. Yet, the question of how equality of opportunity is related to population health outcomes is of substantive interest and practical importance across multiple scientific and policy realms.

The relatively few studies where the health effects of equality of opportunity have been addressed have found that lower equality of opportunity, as measured by intergenerational changes in socioeconomic position, is related to higher mortality rates, poorer mental health, and worse health-related behaviors [7–9]. However, these studies have a relatively narrow geographical focus on Europe or the United States, while almost nothing is known about comparable associations elsewhere in the world.

There are several reasons to believe that equality of opportunity matters for health outcomes in various country settings. Equality of opportunity can enhance individuals’ sense of control over their lives. When people believe they have the ability to influence their own outcomes, they experience better mental and physical health. A sense of control is linked to lower stress levels, healthier behaviors, and better coping strategies [10,11]. High equality of opportunity can foster self-efficacy, which is the belief in one’s ability to achieve goals, and it has been linked to positive health behaviors such as regular exercise, healthy eating, and adherence to medical advice [12,13]. Inequality of opportunity, in turn, can lead to chronic stress and trigger physiological responses that can contribute to cardiovascular diseases, weakened immune function, and other health problems [14].

As individuals constantly compare themselves to others in their social environment [15], in societies with high equality of opportunity, these comparisons are less likely to result in feelings of inferiority, thereby promoting better mental health [16]. In high inequality of opportunity settings, if people perceive a significant gap between their own life chances and those of others, they might experience relative deprivation. This feeling is more prevalent in societies with low equality of opportunity and is linked to negative health outcomes, including higher rates of mental illness and unhealthy behaviors [17]. When individuals believe that opportunities are fairly distributed, they are more likely to feel valued and respected, which enhances their psychological well-being [18]. Conversely, perceived unfairness can lead to feelings of resentment and social distrust, negatively affecting health [19,20]. High equality of opportunity may contribute to social cohesion and trust within communities.

Intergenerational equality of opportunity can be measured in various ways, with much of the literature focusing on economic aspects, such as earnings or occupational mobility. This study examines a different but equally important dimension: the link between parents’ and offspring’s educational attainment [21]. Education is widely recognized as both a key component of equality of opportunity [22] and a major determinant of population health [23]. At the same time, the concept of equality of opportunity is inherently multidimensional and empirically complex. It can encompass fairness in access to education, income, employment, or health, and may be defined in absolute or relative terms. Choosing how to operationalize it involves important trade-offs: while economic mobility indicators may capture material well-being more directly, they are often less available and less comparable across diverse national contexts. Educational mobility, by contrast, offers a widely accessible and policy-relevant proxy that reflects early-life circumstances and institutional structures, albeit not capturing all facets of opportunity. In this study, we focus on educational mobility as one key and measurable dimension of equality of opportunity, while acknowledging the broader landscape of possible definitions.

Drawing on the theoretical framework presented above and prior research that links social mobility, structural opportunity, and population health, we test the following central hypothesis: *Higher levels of intergenerational educational mobility, indicating greater equality of opportunity, are associated with lower all-cause mortality rates*. To test the relationship between equality of opportunity and mortality, we analyze register and survey data from five birth cohorts across 148 countries, adjusting for a wide range of covariates. Covering 96% of the global population, this study expands on previous research by assessing the investigated association on a global scale.

## Methods

### Data

To investigate whether mortality rates are associated with equality of opportunity for consecutive birth cohorts born since the mid-20th century in 148 countries, we utilize data from the World Health Organization (WHO) Mortality Database and the Global Database for Intergenerational Mobility. Our outcome variable is the logged country-level age- and sex-adjusted all-cause mortality rate. The WHO Mortality Database includes deaths recorded in national vital registration systems dating back to 1950. This database contains information on deaths recorded in national vital registration systems. The data represent official national statistics, as provided by the respective countries’ authorities to the WHO. Each WHO member state submits population data, along with mortality data, for the population covered by its death registration system. In countries with incomplete vital registration systems, WHO employs demographic techniques to estimate the completeness of death recording for the given population, enabling the calculation of death rates.

Our key predictor variables, the measures of equality of opportunity, derive from the World Bank’s Global Database on Intergenerational Mobility (GDIM) [24]. Global population coverage in this dataset exceeds 90% in all regions except the Middle East and North Africa, where 81% of the population is covered. Intergenerational mobility in educational attainment is assessed for individuals born in cohorts spanning 10-year intervals from 1940 to 1989. For instance, those born between 1950 and 1959 are categorized as the 1950s cohort, and ‘parents’ refers to the parents of individuals in this cohort. 75% of the data are derived from retrospective data on parental educational attainment [25]. Retrospective questions in surveys explicitly ask all adult respondents about their own education and the education of their parents. This focus was central to identifying the relevant surveys for the study.

To ensure that most respondents from the 1980s cohort had reached an age where their education could be considered complete, surveys conducted since 2006 were included in the GDIM. When multiple surveys with retrospective data were identified, the selection for inclusion in the GDIM was based on factors such as sample size and the availability of detailed categories or years of schooling in educational attainment. For most developing economies outside the Eastern Europe and Central Asia region, as well as the Latin America and Caribbean region, cross-sectional household income or expenditure surveys are utilized. In contrast, social surveys, such as the European Social Survey, the Latinobarómetro Survey, and the Life in Transition Survey, are employed for most economies within the Eastern Europe and Central Asia region and the Latin America and Caribbean region. The full list of employed surveys is shown in the supplementary information (Table S1).

Out of various measures of equality of opportunity in educational attainment given in GDIM, we select four indicators that are operationalized in the following ways: (1) The correlation between parental and child educational attainment, measured in years of education. This measure of equality of opportunity is based on the Pearson correlation coefficient between the years of education of parents and their children. Higher values of the Pearson correlation coefficient for a cohort in a country indicate greater intergenerational persistence and, therefore, lower equality of opportunity; (2) The probability that an individual stemming from the bottom half of the educational distribution ends up in the bottom quartile of that distribution. Higher values of this measure indicate stagnation of life chances of individuals who were initially disadvantaged; (3) The probability that an individual stemming from the top quartile of the educational distribution ends up in the bottom half of that distribution. This measure indicates the extent to which downward intergenerational mobility takes place in society; (4) The probability that an individual stemming from the bottom half of the educational distribution ends up in the top quartile of that distribution. Higher values of this measure indicate greater upward educational mobility and a higher level of equality of opportunity.

### Statistical analyses

Our baseline model is a multilevel random effects model in which we regress age- and sex-adjusted all-cause mortality rates on each measure of educational mobility. This modeling strategy is well-suited to our data structure, where each observation is nested within both countries and birth cohorts. The use of crossed random intercepts allows us to account for unobserved heterogeneity at both levels, such as health system characteristics or cohort-specific historical shocks, that could bias estimates in simpler models. Moreover, unlike fixed-effects approaches, random effects enable us to retain key country-level predictors that are relatively stable over time (e.g. education levels, political freedoms), which are central to our research question. Our intraclass correlation diagnostics indicate substantial clustering by both country and cohort, justifying the multilevel specification.

We then run adjusted models that control for the following country-level covariates that are known to be associated with mortality outcomes with the descriptive statistics presented in Table 1: Mean levels of education, measured in years of completed schooling; the natural logarithm of per capita gross domestic product (GDP); rates of unemployment, measured in percentage of the active labor force; government spending on health as a share (percentage) of GDP; income inequality, as measured by the Gini index, on a scale from 0 to 1; Political freedoms, as measured by the Freedom House Index, on a scale from 0 to 10. Descriptive statistics and the sources of data are shown in Table 1. For all models, we calculate standard errors that are corrected for clustering at both the cohort and country levels. We cluster standard errors at both the cohort and country levels to account for possible non-independence of observations within each dimension. This choice reflects the fact that our key independent variables (educational mobility measures) are defined at the cohort-by-country level, and mortality outcomes are likewise measured along these axes. Clustering on both levels helps mitigate biases in standard errors due to correlated residuals within cohorts (e.g. due to shared global shocks or education trends) and within countries (e.g. due to persistent national characteristics). To present our results, we simulate from the model estimates to visualize conditional all-cause mortality distributions.

**Table 1.** Descriptive statistics.

	Mean	St. dev. (SD)	Min	Max
Log of all-cause mortality rate	6.61	1.41	3.47	10.30
Parent-child educational correlation	0.44	0.12	0.05	0.78
Stagnant educational mobility	0.35	0.07	0.10	0.67
Downward educational mobility	0.25	0.08	0.00	0.56
Upward educational mobility	0.15	0.05	0.02	0.36
Mean years of education	10.90	3.01	0.95	16.58
Log of GDP per capita in USD	9.33	1.13	6.93	11.41
Unemployment rate (%)	8.76	5.72	0.50	29.00
Health spending as a share of GDP (%)	7.63	2.42	3.04	16.82
Income Gini index	0.39	0.08	0.25	0.71
Political freedoms	2.45	1.62	1.00	7.00

*Sources:* The WHO Mortality Database; The World Bank’s Global Database on Intergenerational Mobility (GDIM); The World Development Indicators; The Standardized World Income Inequality Database; Freedom in the World data-set.

## Results

### Main findings

We visualize the estimated conditional all-cause mortality distribution in high- and low-equality of opportunity countries for four measures of intergenerational educational mobility. In what follows, we refer to these four measures as parent-child educational correlations, stagnant educational mobility, downward educational mobility, and upward educational mobility, respectively. We use our parameter estimates and corresponding standard errors to simulate the mortality rate per 100,000 population separately in high- versus low-equality of opportunity countries, while holding all covariates of all-cause mortality constant. We define ‘high’ versus ‘low’ as one standard deviation above versus below the average level of the corresponding measure of equality of opportunity. This threshold provides a clear and interpretable benchmark that captures meaningful variation across countries and cohorts, and it is commonly used in comparative research to illustrate effects across the empirical distribution of continuous variables [26,27]. We then compute the estimated first differences in mortality between high and low equality of opportunity regimes and plot the results. In doing so, we consistently focus on the reduction of mortality associated with greater equality of opportunity. The underlying regression results are reported in the supplementary information, Tables S2-S5.

**Figure 1.**
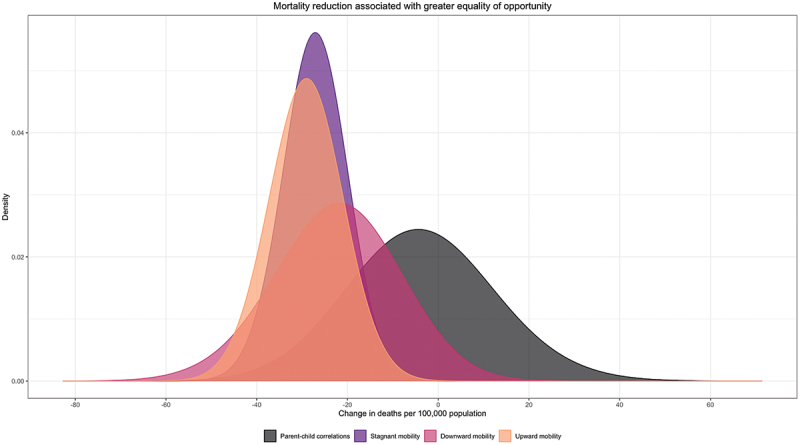
Baseline findings. *Notes:* Model estimates derived from country-level random effects regressions are used to visualize changes in all-cause mortality associated with greater equality of opportunity, as operationalized in four different ways. The model outcome variable is the logged all-cause mortality rate per 100,000 population in all countries; total *N* = 773. Confidence intervals are computed using standard errors clustered at cohort and country levels. The figure shows the difference in the conditional distribution of all-cause mortality rates between high- and low-equality-of-opportunity countries, adjusted for age, sex, mean education, log per capita GDP, unemployment, health spending, income inequality, and political freedoms. The figure shows the net change in the conditional all-cause mortality distribution associated with lower (i.e. a one SD decrease) parent child educational correlations, less stagnant educational mobility, less downward educational mobility, and more upward educational mobility, respectively.

Our baseline findings are visualized in Figure 1, where we present the net reduction in all-cause mortality rates associated with comparing high- to low-opportunity countries after accounting for a range of socio-demographic controls for each of our four measures of equality of opportunity. Parent-child educational correlations are not significantly associated with all-cause mortality (3.6; 95% CI: −33.0, 25.8). However, lower stagnant mobility, as captured by our second measure, is significantly associated with 27.3 fewer all-cause deaths per 100,000 population (95% CI: −40.0, −14.5). Lower downward education mobility, as captured by our third equality of opportunity measure, is associated with 22.5 fewer deaths per 100,000 population (95% CI: −47.6, 2.66), while higher upward educational mobility, as captured by our fourth equality of opportunity measure, is significantly associated with a reduction of 29.1 deaths per 100,000 population (95% CI: −43.8, −14.4).

### Results for high- and low-income countries

**Figure 2.**
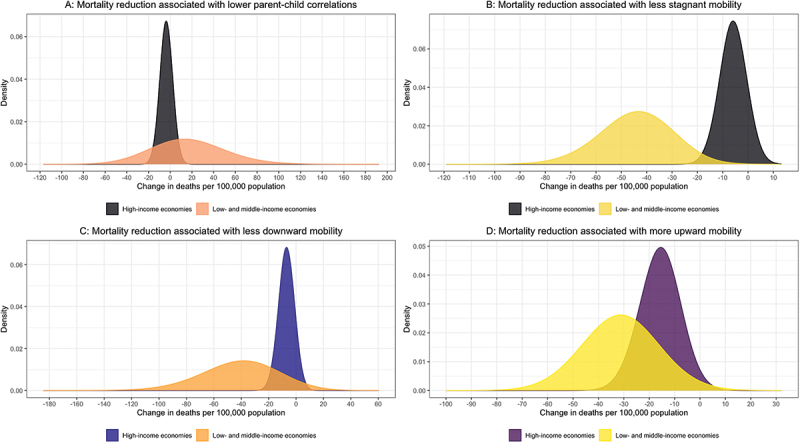
Geographically disaggregated findings. *Notes:* Model estimates derived from country-level random effects regressions are used to visualize change in all-cause mortality associated with greater equality of opportunity, as operationalized in four different ways. Models are run separately for low- and middle-income countries and for high-income countries. Confidence intervals are computed using standard errors clustered at cohort and country levels. The figure shows the difference in the conditional distribution of all-cause mortality rates between high- and low-equality-of-opportunity countries, adjusted for age, sex, mean education, log per capita GDP, unemployment, health spending, income inequality, and political freedoms. Panel A shows the net change in the conditional all-cause mortality distribution associated with lower-parent child educational correlations; panel B shows the corresponding net change associated with less stagnant educational mobility; panel C shows the corresponding net change associated with less downward educational mobility; and panel D shows the corresponding net change associated with higher upward educational mobility.

In a geographically disaggregated analysis, we break down the models by levels of national income. Panel A of Figure 2 shows results for lower parent-child correlations, which in high-income countries are associated with 3.64 fewer all-cause deaths per 100,000 population (95% CI: −14.3, 6.99), compared to 16.6 excess deaths per 100,000 population in low- and middle-income countries (95% CI: −45.1, 78.4), albeit not significantly so. Panel B of the same figure shows that less stagnant mobility is associated with 5.91 fewer deaths per 100,000 population in high-income countries (95% CI: −15.5, 3.71) compared to 44.3 fewer deaths in low- and middle-income countries (95% CI: −70.6, −18.1). Less downward mobility is linked to 6.97 fewer deaths per 100,000 population in high-income countries (95% CI: −17.4, 3.50) compared to 41.3 fewer deaths in low- and middle-income countries (95% CI: −92.4, 9.87), as shown in panel C. Finally, higher upward mobility is associated with 15.8 fewer deaths per 100,000 population (95% CI: −30.2, −1.28) in high-income countries, while in low- and middle-income countries, the corresponding figure is 31.2 (95% CI: −58.8, −3.70), as shown in panel D.

### Gender differences

**Figure 3.**
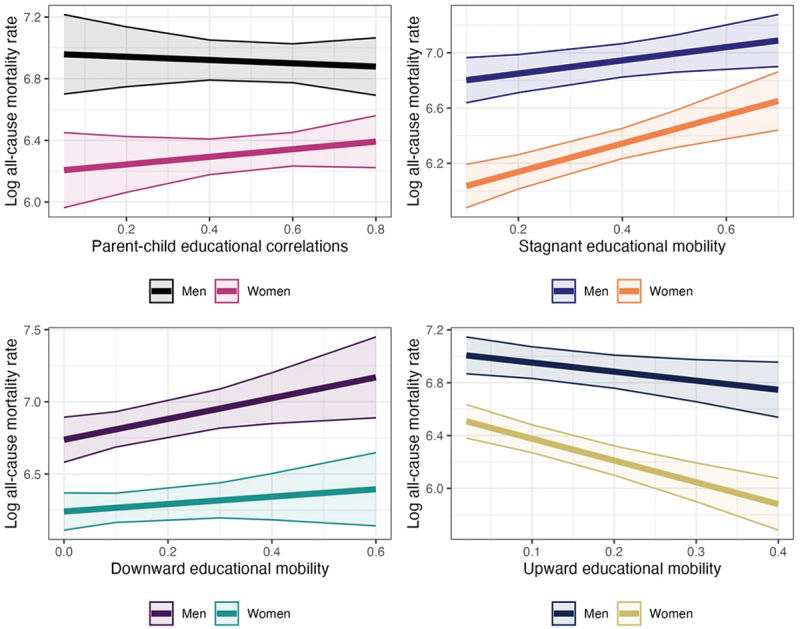
Gender-disaggregated findings. *Notes:* Model estimates derived from country-level random effects regressions are used to visualize change in all-cause mortality associated with the interaction between gender and greater equality of opportunity, as operationalized in four different ways. Confidence intervals are computed using standard errors clustered at cohort and country levels. The figure shows the net association between all-cause mortality rates and each of the four equality of opportunity measures, interacted with sex and adjusted for age, mean education, log per capita GDP, unemployment, health spending, income inequality, and political freedoms.

We also assess the extent to which the association between equality of opportunity and mortality interacts with gender, as visualized in Figure 3. Along with revealing a well-established gender mortality gap of women having better health outcomes and longer life expectancy [28], we find some gender divergences in the association between educational mobility and mortality. For the measures of stagnant educational mobility and upward educational mobility, the association between intergenerational equality of opportunity and log all-cause mortality rates is stronger among women than among men. In the supplementary information, Figures S1 and S2, we also demonstrate the gender-disaggregated analyses separately in high- and low-income countries. The results for these two sets of countries are comparable to the findings reported in the main analyses.

## Discussion

Intergenerational equality of opportunity implies that children should have equal chances for success, regardless of their parents’ socioeconomic status. While considerable research has examined the impact of intergenerational mobility on individual health and well-being [29–31], its effect on population health remains less understood. This study addresses this gap by examining the relationship between equality of opportunity and all-cause mortality across 148 countries, which encompass 96% of the global population. Several mechanisms explain how equality of opportunity may influence health. A greater sense of control over one’s life, fostered by equal opportunities, contributes to better mental and physical health, while unequal opportunities can lead to chronic stress and related health issues. Perceptions of fairness and justice enhance psychological well-being, whereas perceived unfairness can undermine it. By analyzing global data on educational mobility, this study offers new insights into the relationship between equality of opportunity and health outcomes in diverse contexts. This global perspective adds to the understanding that improving social mobility can be an important pathway toward reducing health disparities across both developed and developing nations.

Our findings suggest a significant association between equality of opportunity, measured with educational mobility, and all-cause mortality rates on a global scale. Net of a wide variety of social, economic, and political predictors of health, stagnant equality of opportunity is significantly associated with increased all-cause mortality rates, with each standard deviation change leading to a 6% increase in mortality rates. Conversely, upward educational mobility is significantly linked to lower all-cause mortality rates. The geographically disaggregated analysis reveals consistent patterns across both developed and developing countries, showing a harmful connection between stagnant educational mobility and a beneficial link between upward educational mobility and all-cause mortality. One possible reason why we do not find statistically significant relationships when using the measure of parent-child educational correlation is that the relationship between parents and children’s educational attainment is not linear. The Pearson correlation may fail to capture significant associations if the considered relationship in different country contexts is more complex or nonlinear.

In addition to the psychological mechanisms described in the introduction of this study, in less socially fluid environments, perceptions of inequality of opportunity may discourage individuals from disadvantaged backgrounds from investing more in their health [7]. This discouragement can manifest in behavioral disinvestment, such as reduced motivation to engage in preventive health measures (e.g. regular check-ups, healthy diets, physical activity), as well as in increased likelihood of engaging in harmful coping behaviors, including smoking, substance use, or sedentary lifestyles [32–36]. Prior research suggests that feelings of powerlessness and low personal efficacy associated with structural disadvantage and blocked opportunities can undermine health-promoting behaviors and increase chronic stress exposure, both of which have long-term consequences for morbidity and mortality [37,38]. Thus, perceived inequality of opportunity may operate not only through material constraints but also by shaping expectations and behaviors linked to health investment.

With low equality of opportunity, not anticipating future possibilities may decrease the likelihood that the socioeconomic benefits of being healthier, such as securing a high-quality job with stable employment and a good salary, will materialize [39]. Along with the Spirit Level theory [40] predictions, high inequality of opportunity can significantly impact mortality outcomes. Societies with greater disparities in opportunities face higher mortality rates due to increased stress, reduced social cohesion, and poorer overall health. Furthermore, according to Rising from Rags’ [41] theoretical perspective, equality of opportunity through upward educational mobility can be linked to lower mortality because it promotes a sense of control and agency, leading to better mental health and healthier behaviors. It can also foster social cohesion and supportive communities, enhancing health outcomes. These combined factors create an environment where individuals can lead healthier lives, resulting in lower mortality rates [8].

In addition to the mainstream psychosocial and structural explanations, mechanisms specific to low- and middle-income countries may also shape the observed associations. For example, high levels of informality in labor markets can weaken the protective effect of upward educational mobility if formal qualifications do not reliably translate into secure or well-remunerated employment. Similarly, heterogeneity in education quality, especially between urban and rural areas or across income groups, can result in inflated attainment figures that mask limited real improvements in human capital. In such cases, mobility may not reflect meaningful gains in opportunity or health resilience. Moreover, political instability, under-resourced public health systems, and weak institutional accountability can limit the extent to which any educational advantage translates into better health outcomes. These contextual factors may affect the identified associations observed in some low-income settings, highlighting the need for more thorough, regionally grounded research on the mobility – mortality relationship.

The wider confidence intervals observed for the estimates of low-income countries in our analysis can be attributed to several factors. First, data quality and availability tend to be lower in low-income settings. Many of these countries lack comprehensive and reliable mortality registration systems, leading to greater uncertainty in the data used to estimate mortality rates. Second, socioeconomic and political instability in many low-income regions can introduce fluctuations in health outcomes and mobility patterns, making it difficult to capture stable associations between educational mobility and mortality. Finally, the diverse range of contexts within the low-income category means that any given estimate may be shaped by a broad set of unmeasured factors, contributing to more uncertainty around the central estimates.

The somewhat stronger associations observed among women in our study may reflect gendered patterns in both educational mobility and health vulnerability. In many contexts, upward mobility may confer more substantial social and economic returns for women than for men, especially where female education historically lagged behind. Additionally, women’s health behaviors and outcomes could be more sensitive to structural and psychosocial conditions, such as household stability and perceived life chances. These factors may amplify the health benefits of experiencing upward mobility, or the harms of downward mobility, more markedly among women.

In addition to these structural and contextual challenges, smaller sample sizes in stratified analyses, particularly for gender-disaggregated results, further reduce statistical precision, as reflected in graphically visualized results. While the direction and magnitude of the estimated associations are broadly consistent with theoretical expectations, the wide confidence intervals imply that these results should be interpreted with caution. Rather than offering definitive conclusions, they point to suggestive patterns that merit further investigation. Future research should aim to validate these findings using richer datasets and examine whether gendered or regional mechanisms linking educational mobility and mortality differ systematically across countries and income groups.

There are some other limitations to this study. Given the observational nature of the data, we refrain from making any direct causal claims; however, previous research provides strong reasons to expect a positive causal relationship between equality of opportunity and mortality [6–8,42]. Due to a lack of data and limited statistical power, we are unable to further disaggregate our analysis by world subregions, social class, or cause of death. Mortality rates are the only cross-nationally comparable data, mostly derived from administrative sources, for the available birth cohorts, for whom we also lack more detailed socio-demographic information. Additionally, GDIM coverage is not uniform across the world’s regions. While the database achieves near‑universal coverage globally, coverage is comparatively lower in the Middle East  and  North Africa. Estimates for that region are therefore based on fewer source surveys and should be interpreted with caution.

Although we adjust for a wide array of country-level covariates, other potentially relevant factors are not accounted for due to data limitations. These may include cultural norms related to health behaviors, historical legacies such as colonization or conflict, and institutional characteristics that shape both educational mobility and health systems. To the extent that these unobserved factors covary with both our predictor and outcome measures, omitted variable bias remains a possibility. The multilevel design helps absorb some unmeasured heterogeneity; however, future studies could benefit from incorporating richer contextual data to more precisely estimate the pathways linking equality of opportunity to mortality. Further, although GDIM restricts surveys to those conducted since 2006 to ensure that individuals in the 1980s cohort had completed their education, there may be isolated cases in which educational attainment was still ongoing at the time of the survey. However, given the typical age of survey respondents and the GDIM’s internal quality controls, any resulting bias is likely to be minor and non-systematic.

An additional limitation of our study concerns the possibility of reverse causality: that is, whether population health conditions could influence levels of educational mobility rather than the other way around. While our analysis conceptualizes intergenerational educational mobility as a structural feature of opportunity within a given country-cohort context, shaped by national education systems and broader institutional conditions, it is plausible that poor population health, especially during early childhood, could constrain educational attainment across generations [43]. However, our use of aggregate cohort-level measures rather than individual-level exposures helps mitigate this risk, as it reduces the likelihood that reverse causation at the individual level is driving the observed associations. Moreover, educational mobility is generally established relatively early in life, while our mortality outcomes pertain to adult populations, making it temporally implausible for adult mortality patterns to influence earlier educational structures.

Nevertheless, our analysis corroborates the notion that equality of (educational) opportunity is not only a principle of fairness but has potentially beneficial effects on population health. The finding for upward educational mobility, particularly, highlights the potential magnitude of such beneficial effects. Our overall results thus lend support to policy initiatives that aim to bolster upward intergenerational mobility, such as early life interventions, universal access to early childhood education, affordable higher education, and other investments in human capital, as well as broader welfare policies geared towards equality of opportunity, educational improvement, and expansion [44].

## Conclusion

This study provides novel evidence that intergenerational educational mobility is meaningfully associated with population-level mortality rates across a global sample of 148 countries. Specifically, lower stagnant mobility and higher upward mobility are consistently linked with reduced all-cause mortality, even after adjusting for a wide array of socioeconomic, demographic, and political factors. These findings support our hypothesis that enhancing equality of opportunity, particularly by expanding access to education across generations, can be a powerful mechanism for improving public health. While causality cannot be established, the global consistency of the patterns highlights the importance of integrating policies aimed at promoting social mobility into broader health strategies. Future research should explore these dynamics in more granular detail across world regions and investigate potential causal pathways. In the meantime, policymakers should recognize equality of opportunity not only as a moral and economic imperative but also as a public health priority.

## Supplementary Material

RR_GHA_SI.docx

## Data Availability

The data used in this study are openly available. The analysis code for this study is available in the OSF repository.
