# Supplementary material for: Equality of opportunity and mortality around the world: implications for global public health
Source: Glob Health Action. 2025 Aug 20;18(1):2540167. doi: 10.1080/16549716.2025.2540167 (PMC12372489; doi:10.1080/16549716.2025.2540167)
Supplement: RR_GHA_SI.docx [file ZGHA_A_2540167_SM8271.docx]

**Table S1:** Survey coverage of countries included in GDIM

| **Country** | **Survey** | **Year** | **Co-resident** |
| --- | --- | --- | --- |
| Afghanistan | NRVA | 2011 | Yes |
| Albania | LITS | 2016 | No |
| Angola | IBEP-MICS | 2008 | Yes |
| Argentina | LATINOBAROMETRO | 2015 | No |
| Armenia | LITS | 2016 | No |
| Australia | HILDA | 2015 | No |
| Austria | ESS | 2014 | No |
| Azerbaijan | LITS | 2016 | No |
| Bangladesh | HIES | 2010 | Yes |
| Belarus | LITS | 2016 | No |
| Belgium | ESS | 2014 | No |
| Benin | EMICOV | 2011 | No |
| Bhutan | LSS | 2003 | No |
| Bolivia | EH | 2008 | No |
| Bosnia and Herzegovina | LITS | 2016 | No |
| Botswana | CWIS | 2009 | Yes |
| Brazil | PNAD | 2014 | No |
| Bulgaria | ESS | 2012 | No |
| Burkina Faso | ECVM | 2009 | Yes |
| Cabo Verde | QUIBB | 2007 | Yes |
| Cambodia | CSES | 2012 | Yes |
| Cameroon | ECAM-III | 2007 | Yes |
| Canada | CGSS | 2014 | No |
| Central African Republic | ECASEB | 2008 | Yes |
| Chad | ECOSIT-III | 2011 | Yes |
| Chile | CASEN | 2013 | No |
| China | CFPS | 2012 | No |
| Colombia | ENCV | 2013 | No |
| Comoros | EESIC | 2014 | No |
| Congo, Dem. Rep. | E123 | 2012 | No |
| Congo, Rep. | ECOM | 2011 | Yes |
| Costa Rica | LATINOBAROMETRO | 2015 | No |
| Côte d'Ivoire | ENV | 2008 | Yes |
| Croatia | LITS | 2016 | No |
| Cyprus | ESS | 2012 | No |
| Czech Republic | ESS | 2014 | No |
| Denmark | ESS | 2014 | No |
| Djibouti | EDAM | 2012 | Yes |
| Dominican Republic | LATINOBAROMETRO | 2015 | No |
| Ecuador | ECV | 2013 | No |
| Egypt, Arab Rep. | ELMPS | 2012 | No |
| El Salvador | LATINOBAROMETRO | 2015 | No |
| Estonia | ESS | 2014 | No |
| Ethiopia | LSMS-ISA | 2013 | No |
| Fiji | HIES | 2008 | Yes |
| Finland | ESS | 2014 | No |
| France | ESS | 2014 | No |
| Gabon | EGEP | 2005 | Yes |
| Georgia | LITS | 2016 | No |
| Germany | ESS | 2014 | No |
| Ghana | GLSS | 2012 | No |
| Greece | LITS | 2016 | No |
| Guatemala | LATINOBAROMETRO | 2015 | No |
| Guinea | EIBC | 2012 | Yes |
| Guinea-Bissau | ILAP | 2010 | No |
| Haiti | ECMVM | 2012 | Yes |
| Honduras | EHPM | 2013 | Yes |
| Hungary | ESS | 2014 | No |
| India | IHDS | 2011 | No |
| Indonesia | IFLS | 2014 | No |
| Iraq | IHSES | 2012 | No |
| Ireland | ESS | 2014 | No |
| Italy | ESS | 2014 | No |
| Jamaica | SLC | 2014 | Yes |
| Jordan | HIES | 2010 | Yes |
| Kazakhstan | LITS | 2016 | No |
| Kenya | STEP | 2013 | No |
| Kosovo | LITS | 2016 | No |
| Kuwait | PAPFAM | 2004 | No |
| Kyrgyz Republic | KIHS | 2012 | Yes |
| Lao PDR | STEP | 2012 | No |
| Latvia | ESS | 2014 | No |
| Lebanon | NCLS | 2007 | Yes |
| Lesotho | CMSHBS | 2010 | No |
| Liberia | HIES | 2014 | Yes |
| Lithuania | ESS | 2014 | No |
| Madagascar | ENV | 2010 | Yes |
| Malawi | LSMS-ISA | 2013 | No |
| Malaysia | HIES | 2012 | Yes |
| Mali | LSMS-ISA | 2014 | No |
| Mauritania | EPCV | 2008 | Yes |
| Mauritius | HBS | 2012 | Yes |
| Mexico | MxFLS | 2009 | No |
| Moldova | LITS | 2016 | No |
| Mongolia | HSES | 2012 | Yes |
| Montenegro | LITS | 2016 | No |
| Morocco | ENNS | 2010 | Yes |
| Mozambique | IOF | 2010 | Yes |
| Myanmar | IHLCS | 2010 | Yes |
| Namibia | NHIES | 2009 | Yes |
| Nepal | LSS | 2011 | Yes |
| Nicaragua | EMNV | 2009 | Yes |
| Niger | LSMS-ISA | 2014 | No |
| Nigeria | LSMS-ISA | 2012 | No |
| North Macedonia | LITS | 2016 | No |
| Norway | ESS | 2014 | No |
| Pakistan | PSLM | 2013 | No |
| Panama | ENV | 2008 | Yes |
| Papua New Guinea | HIES | 2009 | Yes |
| Paraguay | EPH | 2013 | Yes |
| Peru | ENAHO | 2013 | Yes |
| Philippines | FIES | 2012 | Yes |
| Poland | ESS | 2014 | No |
| Portugal | ESS | 2014 | No |
| Romania | LITS | 2016 | No |
| Russian Federation | LITS | 2016 | No |
| Rwanda | EICV | 2014 | Yes |
| Senegal | PSF | 2012 | No |
| Serbia | LITS | 2016 | No |
| Sierra Leone | SLIHS | 2011 | Yes |
| Slovak Republic | ESS | 2012 | No |
| Slovenia | ESS | 2014 | No |
| South Africa | NIDS | 2014 | No |
| South Sudan | NBHS | 2009 | Yes |
| Spain | ESS | 2014 | No |
| Sri Lanka | STEP | 2012 | No |
| Sudan | HBS | 2009 | Yes |
| Swaziland | HIES | 2009 | Yes |
| Sweden | ESS | 2014 | No |
| Switzerland | ESS | 2014 | No |
| Taiwan, China | TSCS | 2015 | No |
| Tajikistan | LITS | 2016 | No |
| Tanzania | LSMS-ISA | 2012 | No |
| Thailand | SES | 2012 | Yes |
| Timor-Leste | LSMS | 2007 | No |
| Togo | QUIBB | 2015 | No |
| Tonga | HIES | 2009 | Yes |
| Tunisia | TLMPS | 2014 | No |
| Turkey | LITS | 2016 | No |
| Tuvalu | HIES | 2010 | Yes |
| Uganda | LSMS-ISA | 2014 | No |
| Ukraine | ESS | 2012 | No |
| United Kingdom | ESS | 2014 | No |
| United States | PSID | 2015 | No |
| Uruguay | LATINOBAROMETRO | 2015 | No |
| Uzbekistan | LITS | 2016 | No |
| Vanuatu | HIES | 2010 | Yes |
| Venezuela, RB | LATINOBAROMETRO | 2015 | No |
| Vietnam | STEP | 2012 | No |
| West Bank and Gaza | PECS | 2011 | Yes |
| Yemen, Rep. | HBS | 2014 | Yes |
| Zambia | LCMS-VI | 2010 | Yes |

**Table S2:** Baseline regression estimates

| **Measure** | **Parameter estimate** | **Standard error** | ***p*-value** |
| --- | --- | --- | --- |
| Parent-child correlations | 0.062354 | 0.2361 | 0.79279 |
| Stagnant educational mobility | 0.748317 | 0.17263 | <0.001 |
| Downward educational mobility | 0.49119 | 0.27026 | 0.07512 |
| Upward educational mobility | -1.079755 | 0.27949 | <0.001 |

*Notes:* Covariate-adjusted parameter estimates from country-level random effects regressions for the four different measures of equality of opportunity listed in the left column. The outcome variable is the logged all-cause mortality rate per 100,000 population in all countries; parameter estimates are adjusted for age, sex, mean education, log per capita GDP, unemployment, health spending, income inequality, and political freedoms. Confidence intervals are computed using standard errors clustered at cohort and country levels. Total *N* = 773.

**Table S3:** Regression estimates for low- and middle-income countries

| **Measure** | **Parameter estimate** | **Standard error** | ***p*-value** |
| --- | --- | --- | --- |
| Parent-child correlations | -0.1789 | 0.35486 | 0.6188 |
| Stagnant educational mobility | 0.89593 | 0.24496 | <0.001 |
| Downward educational mobility | 0.66832 | 0.3966 | 0.104 |
| Upward educational mobility | -0.8570 | 0.38878 | 0.0369 |

*Notes:* Covariate-adjusted parameter estimates from country-level random effects regressions for the four different measures of equality of opportunity listed in the left column. The outcome variable is the logged all-cause mortality rate per 100,000 population in low- and middle-income countries; parameter estimates are adjusted for age, sex, mean education, log per capita GDP, unemployment, health spending, income inequality, and political freedoms. Confidence intervals are computed using standard errors clustered at cohort and country levels. Total *N* = 419.

**Table S4:** Regression estimates for high-income countries

| **Measure** | **Parameter estimate** | **Standard error** | ***p*-value** |
| --- | --- | --- | --- |
| Parent-child correlations | 0.0862 | 0.12729 | 0.50434 |
| Stagnant educational mobility | 0.2416 | 0.19956 | 0.238 |
| Downward educational mobility | 0.2284 | 0.17336 | 0.1996 |
| Upward educational mobility | -0.8565 | 0.38907 | 0.0386 |

*Notes:* Covariate-adjusted parameter estimates from country-level random effects regressions for the four different measures of equality of opportunity listed in the left column. The outcome variable is the logged all-cause mortality rate per 100,000 population in high-income countries; parameter estimates are adjusted for age, sex, mean education, log per capita GDP, unemployment, health spending, income inequality, and political freedoms. Confidence intervals are computed using standard errors clustered at cohort and country levels. Total *N* = 354.

**Table S5:** Regression estimates with gender interactions

| **Measure** | **Parameter estimate** | **Standard error** | ***p*-value** |
| --- | --- | --- | --- |
| Parent-child correlations × women | 0.352590 | 0.14712 | 0.02047 |
| Stagnant educational mobility × women | 0.547660 | 0.32960 | 0.10275 |
| Downward educational mobility × women | -0.46269 | 0.21329 | 0.03536 |
| Upward educational mobility × women | -0.966 | 0.33036 | 0.00510 |

*Notes:* Covariate-adjusted parameter estimates from country-level random effects regressions for the four different measures of equality of opportunity interacted with gender, as listed in the left column. The outcome variable is the logged all-cause mortality rate per 100,000 population in high-income countries; parameter estimates are adjusted for age, mean education, log per capita GDP, unemployment, health spending, income inequality, and political freedoms. Confidence intervals are computed using standard errors clustered at cohort and country levels. Total *N* = 773.

**Figure S1:** Gender-disaggregated results in low- and middle-income countries


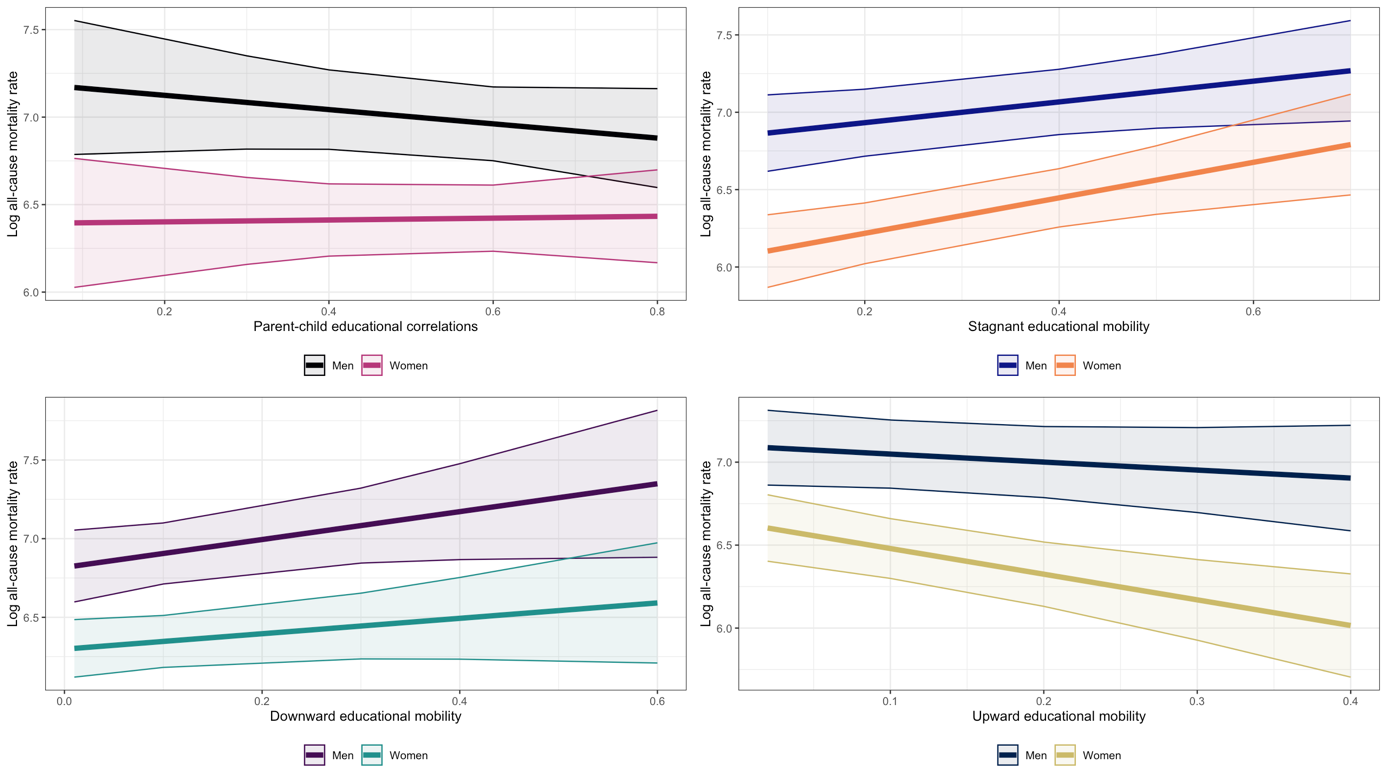


*Notes:* Model estimates derived from country-level random effects regressions are used to visualize change in all-cause mortality associated with the interaction between gender and greater equality of opportunity, as operationalized in four different ways. Confidence intervals are computed using standard errors clustered at cohort and country levels. The figure shows the net association between all-cause mortality rates and each of the four equality of opportunity measures, interacted with sex and adjusted for age, mean education, log per capita GDP, unemployment, health spending, income inequality, and political freedoms.

**Figure S2:** Gender-disaggregated results in high-income countries


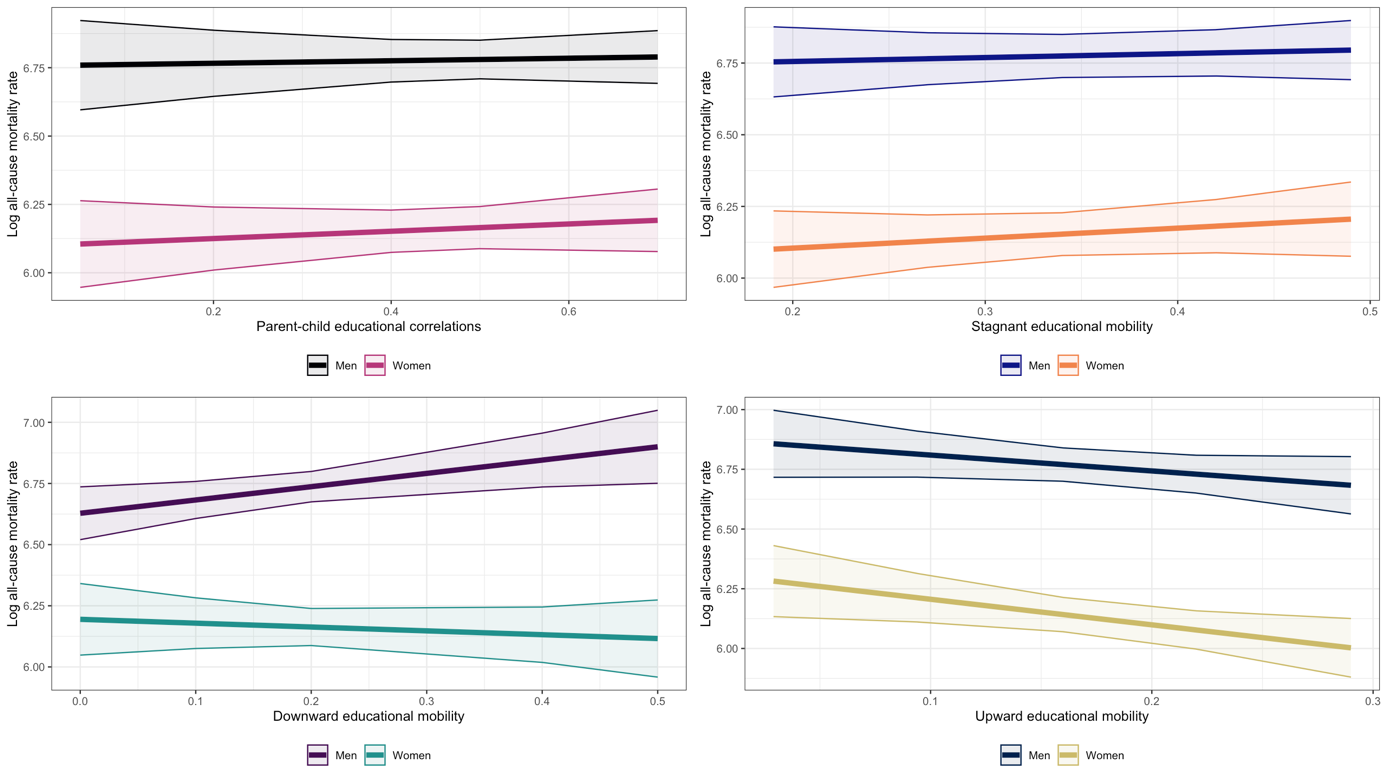


*Notes:* Model estimates derived from country-level random effects regressions are used to visualize change in all-cause mortality associated with the interaction between gender and greater equality of opportunity, as operationalized in four different ways. Confidence intervals are computed using standard errors clustered at cohort and country levels. The figure shows the net association between all-cause mortality rates and each of the four equality of opportunity measures, interacted with sex and adjusted for age, mean education, log per capita GDP, unemployment, health spending, income inequality, and political freedoms.
